# From revascularization to multimodal integrated therapy for peripheral arterial disease: immunomodulation, metabolic correction, and tissue engineering

**DOI:** 10.3389/fimmu.2026.1916929

**Published:** 2026-10-22

**Authors:** Xinyi Tang, Hongcheng Du, Jiaruo Xu, Yuzhen Wang, Yemin Cao, Yongkang Zhang

**Affiliations:** 1 Pan-Vascular Management Center, Shanghai TCM-Integrative Hospital, Shanghai University of Traditional Chinese Medicine, Shanghai, China; 2 Department of Pharmacy, Yiling People’s Hospital of Yichang City, Yichang, Hubei, China; 3 Innovation Research Institute of Traditional Chinese Medicine, Shanghai University of Traditional Chinese Medicine, Shanghai, China

**Keywords:** immunomodulation, macrophage polarization, multimodal integrated therapy, NLRP3 inflammasome, peripheral arterial disease, vascular niche remodeling

## Abstract

Peripheral arterial disease (PAD), a critical manifestation of systemic atherosclerosis (AS), poses substantial therapeutic challenges. Despite pharmacological interventions and revascularization, efficacy remains constrained by high restenosis rates and suboptimal functional recovery. These limitations underscore a necessary transition from localized structural revascularization toward integrated systemic repair, with a particular focus on the underlying immunopathology. The progression of PAD is driven by a dysregulated immune response, characterized by an imbalance in macrophage polarization (M1/M2), overactivation of the NLRP3 inflammasome, and a vicious cycle of immune-metabolic coupling. Current research trajectories emphasize the synergistic orchestration of the vascular microenvironment through immunomodulation. Beyond modulating localized inflammatory responses, emerging strategies target metabolic homeostasis and advanced tissue engineering, including spatiotemporal growth factor delivery, extracellular vesicle-based therapies, and bioengineered scaffolding systems. These interdisciplinary developments point toward a multimodal integrated therapeutic strategy for PAD. Such a strategy would build on conventional revascularization and may address its limitations, including restenosis, through targeted immunomodulation and local tissue regeneration. Whether this approach can improve long-term outcomes compared with revascularization alone remains to be tested. If validated, it could shift the clinical standard beyond vessel patency toward comprehensive, long-term vascular health.

## 1 Introduction

As global demographic shifts accelerate, cardiovascular diseases (CVDs) remain the preeminent challenge to global public health, accounting for an estimated 17.9 million deaths annually, approximately 32% of total global mortality. Within this epidemiological landscape, peripheral arterial disease (PAD) serves as a critical manifestation of systemic atherosclerosis and a pivotal clinical indicator for future major adverse cardiovascular events (MACE). PAD is associated with a high systemic atherosclerotic burden and predicts future major adverse cardiovascular events (1). As of 2021, approximately 238 million people worldwide were diagnosed with PAD (2), with a landmark 2025 forecast predicting a 220% increase in total PAD cases by 2050 (3).

Pathologically, PAD manifests in the lower extremities through progressive arterial wall remodeling, beginning with endothelial dysfunction, followed by lipid accumulation and localized inflammation, and culminating in atherosclerotic plaque formation (**Figure 1**). The pathophysiology involves not only mechanical occlusion but also immune-inflammatory mechanisms. This review discusses the immunopathology of PAD and how emerging multimodal therapies that integrate immunomodulation, metabolic regulation, and tissue engineering may improve its management.

**Figure 1:**
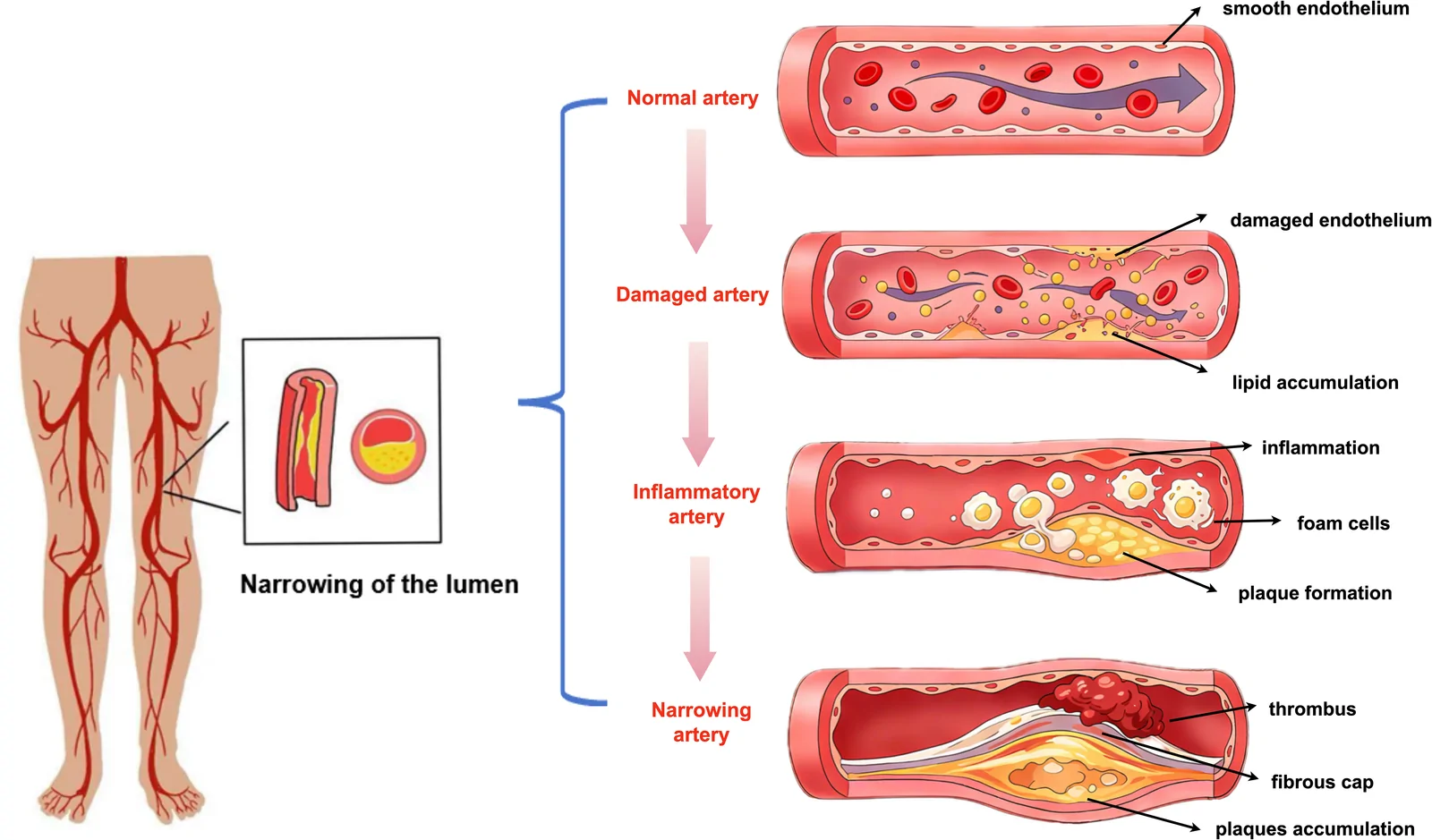
The occurrence and manifestation of PAD. Normal arteries have a smooth endothelium. When damage to the endothelium occurs, lipid accumulation and inflammation are triggered, leading to the formation of foam cells, which subsequently develop into atherosclerotic plaques with fibrous caps. Progressive plaque accumulation leads to significant narrowing of the arterial lumen. The final stage shows the potential complication of thrombosis (clot) formation at the site of the plaque, which can acutely and completely block blood flow. PAD, Peripheral Arterial Disease.

## 2 Immunomodulatory therapies: reprogramming the pro-inflammatory niche toward vascular repair

There are three kinds of inflammation in formation and development of AS: biological, immunological and chemical inflammation. Biological inflammation is the inflammation of the blood vessel wall caused by a variety of pathogens and extravascular infection. Immunological inflammation is related to the activation of immune cells by autoantigen-antibody complexes and the injury of vascular endothelial cells (VECs). A range of bioactive mediators—including lipids, angiotensin II, aldosterone, fibrinogen, advanced glycation end products (AGEs), homocysteine, uric acid, endothelin-1 (ET-1), and resistin—have been shown to exert pro-inflammatory effects on the vasculature (4). Recent studies have revealed an immune axis centered on the innate immune system that operates throughout plaque formation and restenosis after intervention. The key targetable nodes are macrophage M1/M2 imbalance and NLRP3 inflammasome overactivation, which drive chronic inflammation and vascular restenosis. Therefore, the emerging treatment strategy is shifting from the traditional revascularization to the more fundamental modulation of the immune microenvironment. This paradigm aims to reprogram the lesion microenvironment from a pro-inflammatory and pro-stenotic state to an anti-inflammatory and pro-repair homeostasis by selectively interfering with a core and targetable immune axis — namely macrophage M1/M2 imbalance and NLRP3 inflammasome overactivation, it’s the latter driving chronic inflammation and vascular restenosis.

### 2.1 Reprogrammed macrophage polarization: switching from M1 to M2 phenotype

Present data suggest that the ratio of pro-inflammatory M1 and anti-inflammatory M2 macrophages, rather than the number of macrophages, within the AS plaques may determine the progression and regression of AS (5). The ratio of M1 to M2 varies at different stages and locations of AS. Plaques tend to stabilize when M2 macrophages predominate; plaques stability decreases when M1 macrophages infiltrate.

In contrast, the main functions of M2 macrophages are to inhibit inflammation, clear cell debris and apoptotic cells, and promote tissue repair and fibrosis. They are activated by IL-4 and nterleukin-13 (IL-13), secrete anti-inflammatory factors such as IL-10 and TNF-β, and express chemokines such as C-C motif chemokines (CCL) 17, CCL22 and CCL24, thereby initiating functional anti-inflammatory regulatory mechanisms to counteract the chronic inflammatory response caused by M1 macrophages (6).

Kalisadin is an anti-inflammatory plasma protein that inhibits macrophage polarization to the M1 phenotype and promotes M2 macrophage polarization through Kruppel-like Factor 4 (KLF4) activation (7). Studies have shown that myeloid-derived CD147 increases the infiltration and polarization of macrophages, leading to enhanced apoptosis and impaired efferocytosis within plaques. It was then concluded that anti-human CD147 antibody inhibits inflammation and enhances cell proliferation by inhibiting the polarization of M1 macrophages and promoting the transformation of M2 macrophages (8).

Immunomodulatory therapies have been proposed for the prevention of atherosclerotic cardiovascular disease (9). Regulatory T cells (Tregs) are potential tools for the prevention and treatment of atherosclerotic cardiovascular disease by regulating the polarization of macrophages to the M2 phenotype (10). Tregs can secrete cytokines and stimulate macrophage differentiation through mechanisms other than T helper (Th) 1 and Th2. For example, anti-inflammatory cytokines secreted by Tregs, such as IL-10, promote the polarization of M1 macrophages to M2 macrophages (11). Pentoxifylline, a drug that inhibits the Th1 differentiation pathway, induces IL-10 production, blocks Th1 polarization and promotes Th2 polarization, thereby reducing atherosclerotic plaques by approximately 60% in ApoE^-^/^-^ mice (12). PPP1R3B promotes M2-type macrophage polarization and has been associated with reduced atherosclerotic burden in preclinical models (13).

Notably, Traditional Chinese Medicine (TCM) can inhibit the polarization of M1 macrophages and promote the activation of M2 macrophages, thereby delaying the development of AS and enhancing plaque stability. Preclinical studies have demonstrated that certain herbal formulas alleviate atherosclerosis by rebalancing M1/M2 macrophage polarization and dampening local inflammation. The herbal monomer geniposide induced M2 polarization in plaques via upregulation of CXCL14 (14). The herbal extract Leech Peptide HE-D inhibited macrophage migratory activity and promoted macrophage transformation from M1 to M2 via the NF-κB signaling pathway (15). The herbal compound Jisil Haebaek Gyeji-Tang inhibits polarization of inflammatory M1-type macrophages by inhibiting MAPK and NF-κB signaling pathways (16). Curcumin, an active ingredient in curcuma rhizomes, inhibits M1 macrophage polarization and the production of pro-inflammatory factors via TLR4/MAPK/NF-κB pathways, and induces M2 macrophage polarization via activating PPARγ, contributing to an anti-atherosclerosis effect (**Figure 2**) (17).

**Figure 2:**
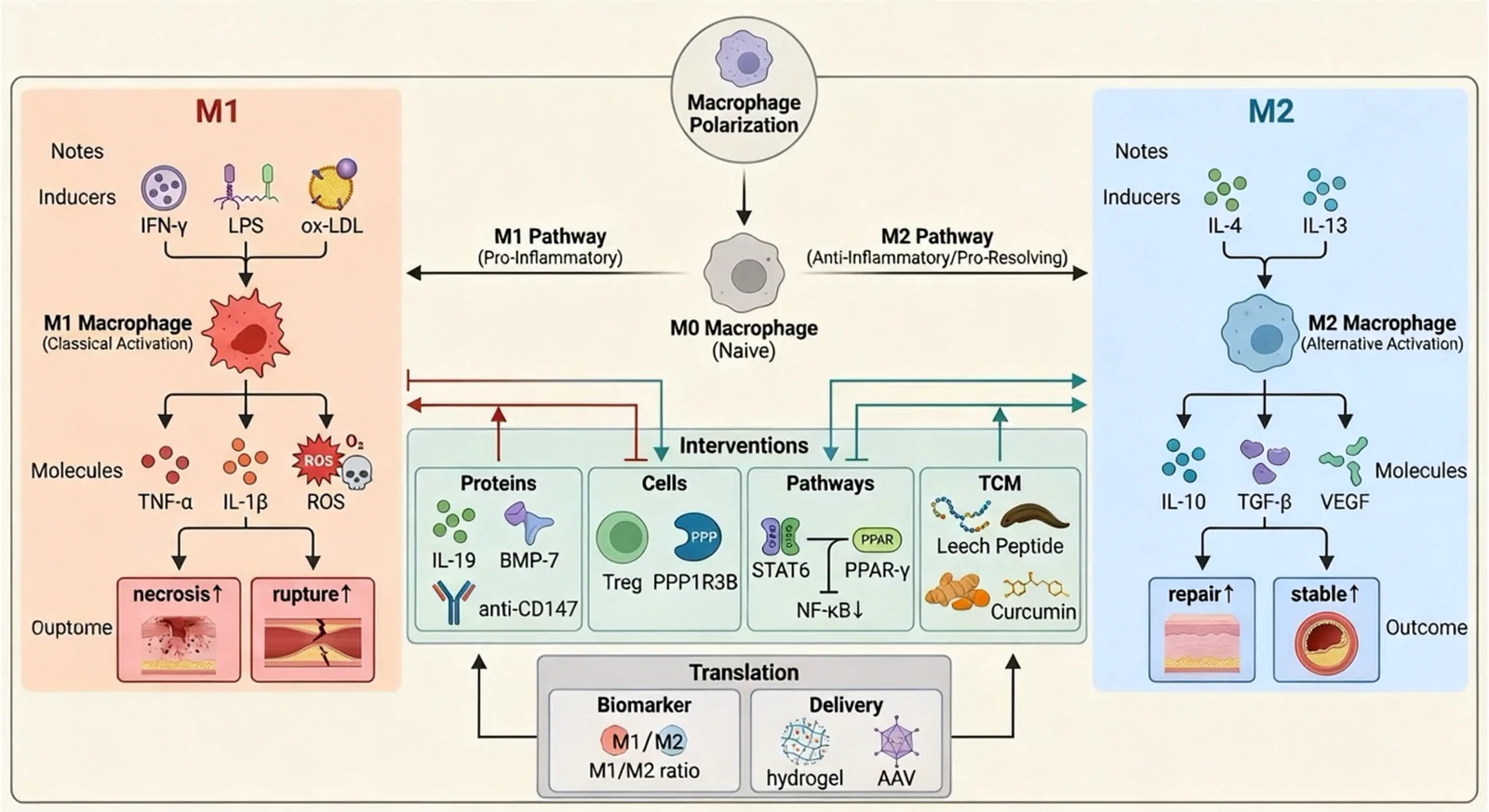
The mechanism, balance regulation and potential intervention strategies of M1/M2 polarization of macrophages in PAD. Macrophages can be classically activated (M1) by stimuli such as IFN-γ, LPS, and ox-LDL, leading to a pro-inflammatory state characterized by the secretion of TNF-α, IL-1β, and ROS, which promote plaque progression and instability. Conversely, alternative activation (M2) driven by signals like IL-4 and IL-13 results in an anti-inflammatory and pro-resolving phenotype, secreting IL-10 and TGF-β to enhance tissue repair and plaque stabilization. The M1/M2 balance is a critical biomarker for disease severity. Key regulatory nodes (e.g., Treg cells, STAT6, NF-κB, PPAR-γ) and emerging intervention strategies, including drugs, natural compounds (e.g., curcumin), and novel delivery systems (e.g., AAV vectors), are available to rebalance this polarized equilibrium to a stable, M2-dominant state. This rebalancing represents a potential therapeutic avenue for PAD. AAV, Adeno-Associated Virus; BMP-7, Bone Morphogenetic Protein 7; CD147, Cluster of Differentiation 147; IFN-γ, Interferon-gamma; IL-1β, Interleukin-1 beta; IL-4, Interleukin-4; IL-10, Interleukin-10; IL-13, Interleukin-13; IL-19, Interleukin-19; LPS, Lipopolysaccharide; M0, Naive Macrophage; M1, Classically Activated Macrophage; M2, Alternatively Activated Macrophage; NF-κB, Nuclear Factor kappa-B; O2, Superoxide anion; ox-LDL, Oxidized Low-Density Lipoprotein; PAD, Peripheral Arterial Disease; PPAR, Peroxisome Proliferator-Activated Receptor; PPAR-γ, Peroxisome Proliferator-Activated Receptor gamma; PPP1R3B, Protein Phosphatase 1 Regulatory Subunit 3B; ROS, Reactive Oxygen Species; STAT6, Signal Transducer and Activator of Transcription 6; TCM, Traditional Chinese Medicine; TGF-β, Transforming Growth Factor-beta; TNF-α, Tumor Necrosis Factor-alpha; Treg, Regulatory T cells; VEGF, Vascular Endothelial Growth Factor.

### 2.2 NLRP3 inflammasome inhibition: targeting the fulcrum of the inflammatory cascade

In AS, the NLRP3 inflammasome links lipid metabolism disorders to chronic vascular inflammation (18). After endothelial cells are stimulated by ox-LDL, ROS and other factors, the NLRP3 inflammasome is activated, releasing IL-1β/IL-18 and inducing pyroptosis, a form of programmed cell death. This promotes the expression of adhesion molecules and chemokines and disrupts endothelial barrier function. HMGB1, in turn, activates the NF-κB signaling pathway, leading to an increased secretion of additional inflammatory mediators, which indirectly contributes to endothelial barrier dysfunction. Pyroptosis mediated by NLRP3 has emerged as a significant contributor to endothelial cell death (19, 20).

In the microenvironment of atherosclerotic plaques, continuous activation of the NLRP3 inflammasome by local triggers (such as ox-LDL and cholesterol crystals) drives a severe inflammatory cascade, including cellular pyroptosis and the massive release of cytokines (20–26). This chronic over-activation serves as a critical pathogenic switch that transitions stable plaques into vulnerable ones, making the NLRP3 pathway a prime target for multimodal integrated therapies (**Figure 3**).

**Figure 3:**
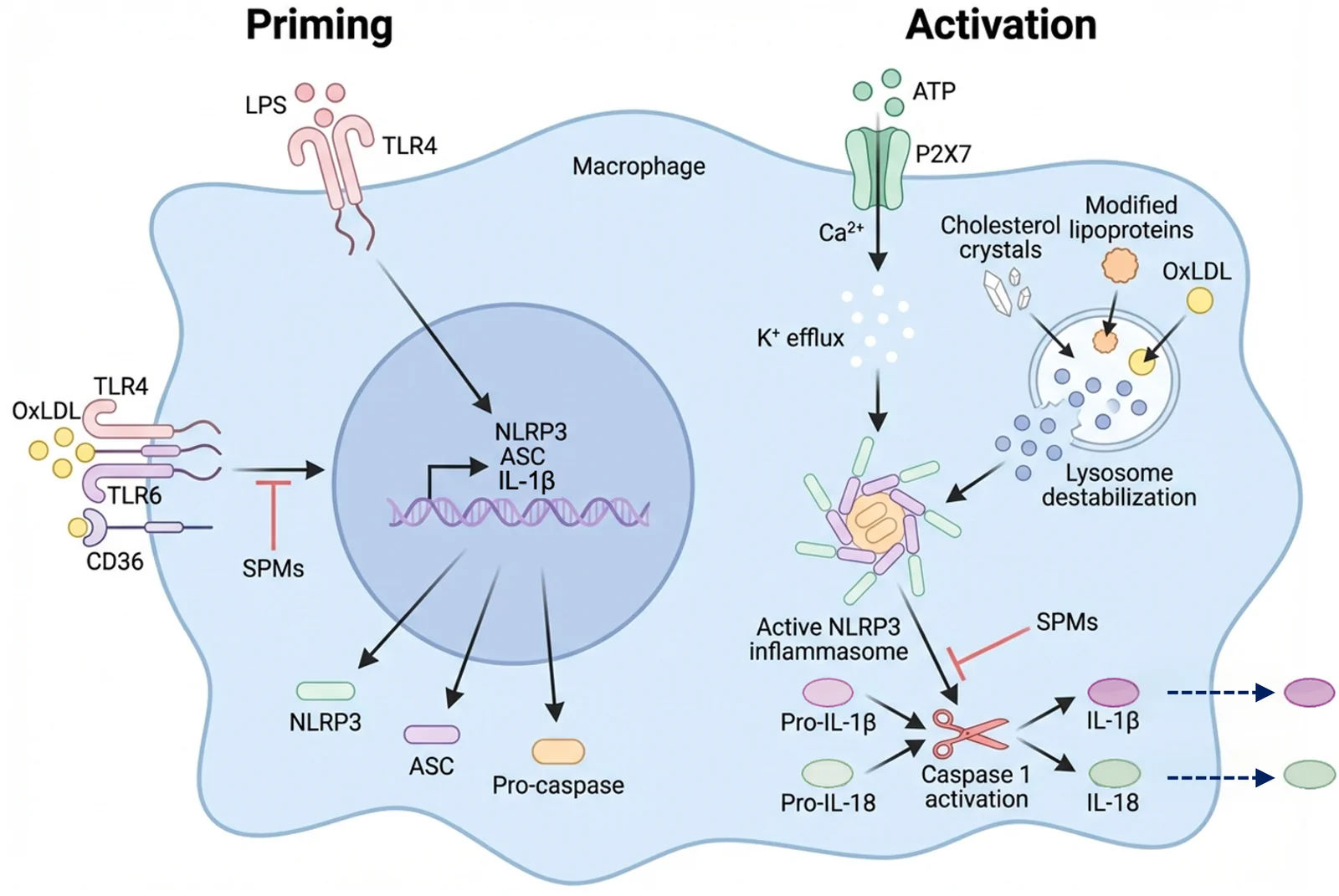
Activation mechanism of the NLRP3 inflammasome in PAD. The NLRP3 inflammasome is a key driver of inflammation in atherosclerotic plaques. A two-step process of NLRP3 inflammasome activation within macrophages, with the priming step (left) initiated by pathogen or damage-associated molecular patterns (PAMPs/DAMPs), such as ox-LDL or LPS, which bind to pattern recognition receptors (such as TLR4). This signal up-regulates the expression of NLRP3 and Il-1β precursors through the NF-κB pathway. The activation step (right) is triggered by a second signal from various atherogenic crystals (for example, cholesterol crystals) or extracellular ATP, leading to K^+^ efflux or lysosomal instability. These events promote the oligomerization of NLRP3 and the assembly of the active inflammasome complex, which recruits and clears procaspase-1 to active caspase-1. Subsequently, pro-Il-1β and pro-IL-18 are cleaved by caspase-1 to mature active forms (IL-1β, IL-18), which secrete IL-1β and IL-18 to promote local and systemic inflammatory responses, thereby exacerbates plaque progression and instability. ASC, Apoptosis-associated speck-like protein containing a CARD; ATP, Adenosine Triphosphate; Ca2+, Calcium ion; Caspase-1, Caspase-1; CD36, Cluster of Differentiation 36; DAMPs, Damage-associated molecular patterns; IL-1β, Interleukin-1 beta; IL-18, Interleukin-18; K+, Potassium ion; LPS, Lipopolysaccharide; NF-κB, Nuclear Factor kappa-B; NLRP3, NOD-like receptor family pyrin domain containing 3; ox-LDL, Oxidized Low-Density Lipoprotein; P2X7, P2X purinoceptor 7; PAD, Peripheral Arterial Disease; PAMPs, Pathogen-associated molecular patterns; Pro-caspase-1, Pro-caspase-1; Pro-IL-1β, Pro-Interleukin-1 beta; Pro-IL-18, Pro-Interleukin-18; SPMs, Specialized Pro-resolving Mediators; TLR4, Toll-like Receptor 4; TLR6, Toll-like Receptor 6.

In recent years, efforts to inhibit the NLRP3 inflammasome have facilitated the discovery and development of multiple NLRP3 inhibitors. These works have focused on the regulation of NLRP3 protein transcription, translation, and activation. Small molecule inhibitors are considered to be a direct and ideal method for the treatment of AS (**Table 1**).

**Table 1:** NLRP3 inflammasome small-molecule inhibitor.

Name of compound	Direct target	Mechanism of inhibition	Development stage
MCC950	NACHT	MCC950 directly interacts with the Walker B motif in the NACHT domain of NLRP3 to block ATP hydrolysis and suppress NLRP3 activation (27, 28).	Preclinical (mouse model of AS)
JT002	NACHT	JT002 allosterically blocks ATP binding and inhibits NLRP3 self-oligomerization (29).	No cardiovascular models reported
CY-09	NACHT	CY-09 directly interacts with the Walker A motif in the NACHT domain of NLRP3 to block ATP hydrolysis and suppress NLRP3 activation (30).	Preclinical (ApoE^−/−^ mice model of aortic valve calcification)
OLT1177	NACHT	OLT1177 directly binds to NLRP3 protein, especially its NACHT domain, and specifically inhibits its ATPase activity (31).	Preclinical (ox-ldl-induced inflammation in macrophages)
Oridonin	NACHT	Oridonin blocks the NLRP3-Nek7 interaction with cysteine residue 279 within the NACHT domain (32).	Preclinical (mouse model of AS)
Tranilast	NACHT	Tranilast binds directly to the NACHT domain of NLRP3 and inhibits its assembly by blocking NLRP3 oligomerization (33).	Preclinical (mouse model of AS)
JC124	NACHT	JC124 directly binds to NLRP3 protein and selectively inhibits the oligomerization of the adaptor protein ASC (34).	Preclinical (mouse model of Acute myocardial infarction)
H28	NACHT	H28 compounds can directly bind to NLRP3 protein and effectively inhibit the assembly and activation of NLRP3 inflammasome (35).	Preclinical (No cardiovascular models reported)
Tabersonine	NACHT	Tab directly bound to NLRP3 NACHT domain, inhibited NLRP3 oligomerization, and then suppressed the activation of NLRP3 inflammasome (36).	Preclinical (mouse model of AS)
Brazilin	NACHT	Brazilin specifically inhibits NACHT to block NLRP3 activation (37).	Preclinical (ex vivo Myocardial ischemia-reperfusion injury model)
INF39	ATPase	INF39 exerts its anti-inflammatory activity by suppressing NLRP3 ATPase activity and the subsequent NLRP3 oligomerization and NLRP3 inflammasome assembly (38).	No cardiovascular models reported
BOT-4-one	ATPase	Alkylation of NLRP3 by BOT-4-one leads to impaired atpase activity of NLRP3, which hinders the assembly of the NLRP3 inflammasome (39).	No cardiovascular models reported
MNS (3,4-methylenedioxy-β-nitrostyrene)	NBD and LRR	MNS specifically prevented NLRP3-mediated ASC speck formation and oligomerization without blocking potassium efflux induced by NLRP3 agonists (40).	No cardiovascular models reported

NACHT, An Acronym for NAIP, CIITA, HET-E, and TP1; NLRP3, NOD-Like Receptor Family Pyrin Domain Containing 3; ATP, Adenosine Triphosphate; NEK7, NIMA-Related Kinase 7; ASC, Apoptosis-Associated Speck-Like Protein Containing a Caspase Recruitment Domain; NBD, Nucleotide-Binding Domain; LRR, Leucine-Rich Repeats; AS, Atherosclerosis; ApoE−/−, Apolipoprotein E Knockout; ox-LDL, Oxidized Low-Density Lipoprotein.

## 3 Breaking the immune-metabolic-ischemic vicious cycle in PAD

### 3.1 Regulation of immune-metabolic coupling

In the pathological process of PAD and its complications, metabolic disorders and immune inflammation do not exist independently, but constitute a core axis of close interaction and mutual amplification, that is, “immune-metabolic coupling”. As the core driving force of metabolic disorders, chronic hyperglycemia directly induces oxidative stress and the generation of AGEs, which damages vascular endothelial function. On the other hand, these metabolic abnormalities directly activate the innate immune system, such as the NLRP3 inflammasome, and trigger sustained release of inflammatory factors. At the same time, the activation of immune cells and inflammatory environment further aggravate insulin resistance and oxidative damage, forming a vicious cycle.

Chronic hyperglycemia leads to oxidative stress, the formation of AGEs, and endothelial damage; diabetes enhances the activity of NLRP3 inflammasome and promotes the secretion of IL-1β and interleukin-18 (IL-18), thereby exacerbating vascular wall inflammation; insulin resistance in diabetic patients impairs the VEGF signaling pathway and inhibits angiogenesis in ischemic tissues. These factors jointly promote the development of PAD. In diabetic vessels, chronic hyperglycemia drives the massive overproduction of reactive oxygen species (ROS) and impairs endogenous antioxidants, leading to severe oxidative stress and eNOS uncoupling (41–45) (**Figure 4**).

**Figure 4:**
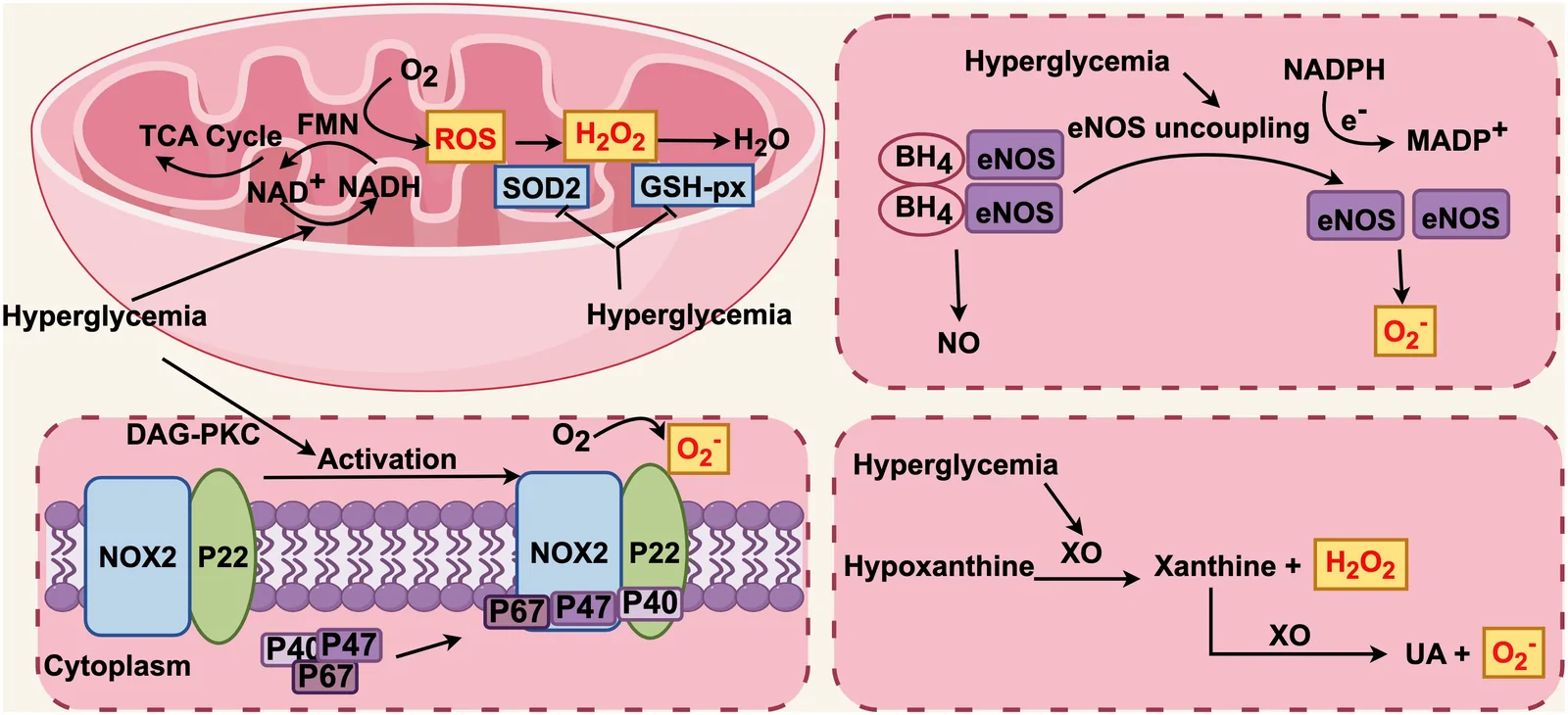
Hyperglycemia-induced oxidative stress in vascular cells. Under hyperglycemic conditions, multiple enzymatic sources contribute to ROS overproduction. In mitochondria, increased flux through the TCA cycle elevates NADH levels, leading to electron leakage at the ETC and generating O_2_−. This is subsequently dismutated to H_2_O_2_ by SOD_2_ and to water by GSH-px. Hyperglycemia also induces eNOS uncoupling by depleting BH_4_, shifting eNOS from NO production to O_2_− generation. Additionally, hyperglycemia activates the DAG-PKC pathway, promoting the assembly and activation of the NOX complex (NOX_2_, P22, P47, P67, P40) and subsequent O_2_− production. Finally, hyperglycemia activates XO, which catalyzes the conversion of hypoxanthine to xanthine and UA, releasing O_2_− and H_2_O_2_. BH_4_, Tetrahydrobiopterin; DAG-PKC, Diacylglycerol-Protein Kinase C; eNOS, Endothelial Nitric Oxide Synthase; ETC, Electron Transport Chain; FMN, Flavin Mononucleotide; GSH-px, Glutathione Peroxidase; H_2_O_2_, Hydrogen Peroxide; NAD+, Nicotinamide Adenine Dinucleotide; NADH, Reduced Nicotinamide Adenine Dinucleotide; NADP+, Nicotinamide Adenine Dinucleotide Phosphate (oxidized); NADPH, Nicotinamide Adenine Dinucleotide Phosphate; NO, Nitric Oxide; NOX, NADPH Oxidase; NOX2, NADPH Oxidase 2; O2-, Superoxide anion; P22, p22phox; P40, p40phox; P47, p47phox; P67, p67phox; ROS, Reactive Oxygen Species; SOD2, Superoxide Dismutase 2; TCA, Tricarboxylic Acid; UA, Uric Acid; XO, Xanthine Oxidase.

Concurrently, the accumulation of advanced glycation end products (AGEs) and their persistent activation of the RAGE receptor trigger a vicious cascade of endothelial dysfunction, vascular stiffness, and accelerated foam cell formation (46–48) (**Figure 5**). Collectively, this combined destruction mediated by oxidative stress and the AGE-RAGE axis forms the primary pathological foundation of diabetic vascular lesions, highlighting why simultaneously targeting these metabolic-inflammatory pathways is an essential component effective multimodal integrated therapy.

**Figure 5:**
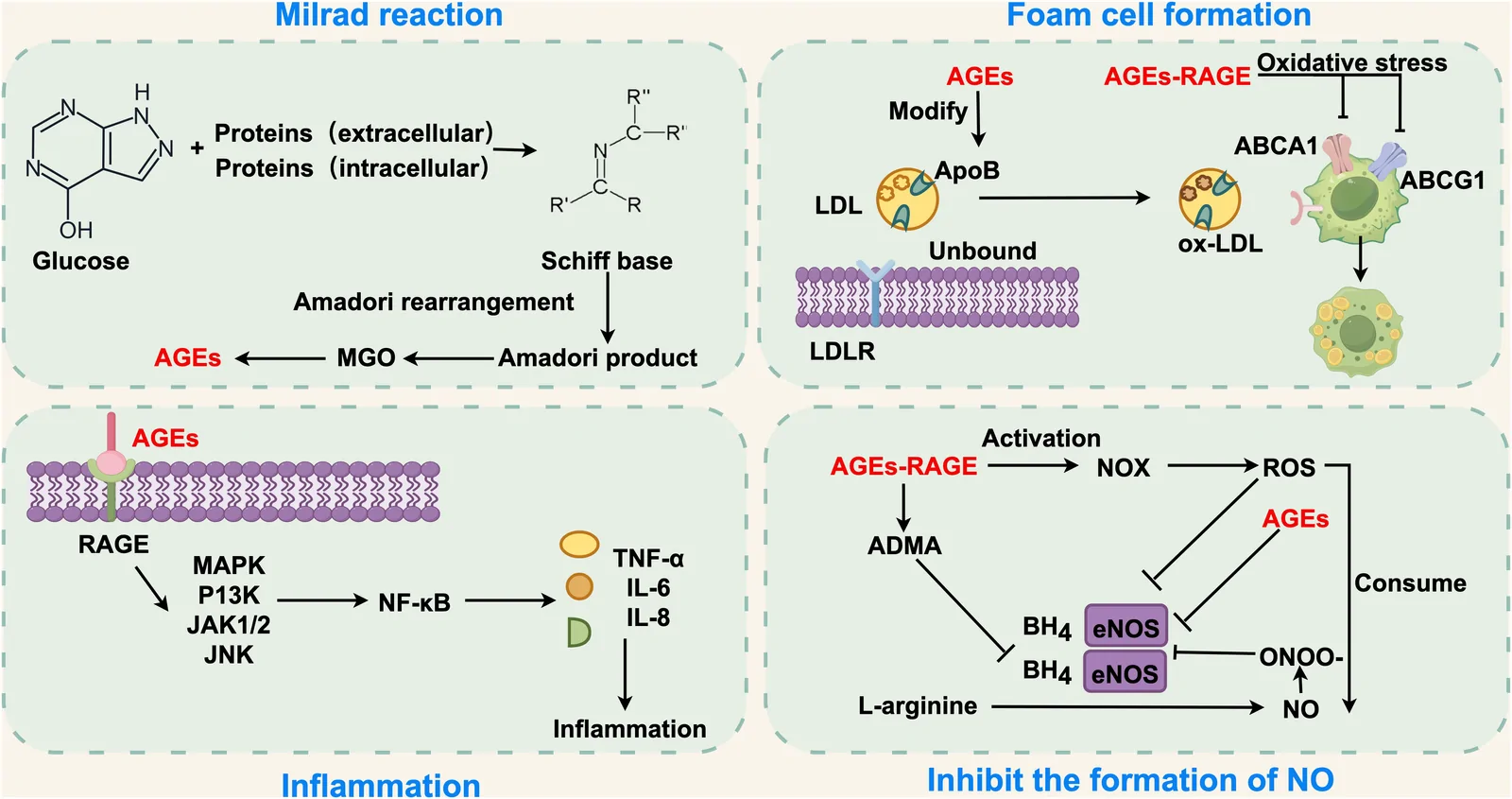
Formation of AGEs and their downstream pathological effects in PAD. Chronic hyperglycemia drives the Maillard reaction, where glucose reacts with extracellular and intracellular proteins to form reversible Schiff bases. These undergo Amadori rearrangement to form Amadori products, which further react via MGO to generate irreversible AGEs. AGEs promote PAD through three main pathways: (1) Foam cell formation: AGEs modify LDL to form ox-LDL. The AGEs-RAGE interaction induces oxidative stress and downregulates ATP-binding cassette transporters ABCA1 and ABCG1, impairing cholesterol efflux and promoting foam cell formation. (2) Inflammation: AGEs-RAGE signaling activates MAPK, PI3K, JAK1/2, and JNK pathways, leading to NF-κB activation and the secretion of pro-inflammatory cytokines (TNF-α, IL-6, IL-8), exacerbating vascular inflammation. (3) Inhibition of NO formation: AGEs-RAGE interaction activates NOX to produce ROS, which consumes NO to form ONOO−. Additionally, AGEs-RAGE signaling induces ADMA, which competes with L-arginine and depletes BH4, leading to eNOS uncoupling and reduced NO production. ABCA1, ATP-Binding Cassette Transporter A1; ABCG1, ATP-Binding Cassette Transporter G1; ADMA, Asymmetric Dimethylarginine; AGEs, Advanced Glycation End Products; ApoB, Apolipoprotein B; BH4, Tetrahydrobiopterin; eNOS, Endothelial Nitric Oxide Synthase; IL-6, Interleukin-6; IL-8, Interleukin-8; JAK1/2, Janus Kinase 1/2; JNK, c-Jun N-terminal Kinase; LDL, Low-Density Lipoprotein; MAPK, Mitogen-Activated Protein Kinase; MGO, Methylglyoxal; NF-κB, Nuclear Factor kappa-B; NO, Nitric Oxide; NOX, NADPH Oxidase; ONOO-, Peroxynitrite; ox-LDL, Oxidized Low-Density Lipoprotein; PAD, Peripheral Arterial Disease; PI3K, Phosphatidylinositol 3-Kinase; RAGE, Receptor for Advanced Glycation End Products; ROS, Reactive Oxygen Species; TNF-α, Tumor Necrosis Factor-alpha.

### 3.2 Comprehensive microcirculation remodeling

Immune regulation aims to terminate inflammatory destruction. The ischemia-reperfusion injury triggers a cascade of complement activation, neutrophil infiltration, and cytokine storm, creating a destructive, amplifying and self-perpetuating cycle of inflammation. Therefore, to achieve effective microcirculation remodeling, the first task is to actively intervene in this immunopathological process. Targeted immune regulation to terminate inflammatory destruction is a key prerequisite for creating a permissive microenvironment for subsequent vascular repair and stability. In PAD, ischemia-reperfusion (I/R) injury is an important cause of tissue injury and poor long-term prognosis after revascularization. Recent studies have confirmed that the complement system is activated in this process, and it produces C3a, C5a and membrane attack complex (MAC) through the classical pathway (IgM-natural antibody-C1q) or alternative pathway, and then stimulates macrophages and neutrophils to release ROS, IL-1β and other inflammatory mediators, which aggravates microvascular injury and limb dysfunction. Therefore, inhibition of complement components has been proposed as a new strategy for the treatment of PAD ischemia (49). So far, research on complement inhibition for ischemia-reperfusion (I/R) injury has drawn largely on solid-organ models, with kidney transplantation being the most prominent example. In preclinical renal studies, agents such as C1 esterase inhibitors (C1-INH) or membrane-anchored CR1 (Mirococept) have been shown to reduce local complement deposition and tubular injury. Extrapolating these observations to peripheral ischemia, however, requires considerable caution. Of note, the anti-C5 monoclonal antibody eculizumab failed to meet its primary endpoint in large randomised controlled trials aimed at preventing delayed graft function in renal transplantation (50, 51). Rather than providing evidence of therapeutic benefit, this clinical disappointment highlights a clear translational gap. Although complement blockade remains conceptually attractive for mitigating I/R injury, the lack of efficacy with systemic inhibition in the transplant setting suggests that any proposed advantage for microvascular thrombosis in PAD is, at present, purely speculative. Future work should therefore focus on PAD-specific vascular models before any clinical conclusions can be drawn.

Direct correction of metabolic derangement in ischemic tissue is the basis for repair. The generation of ROS and AGES can be reduced at the source by controlling HbA1c within the ideal range through lifestyle intervention, oral hypoglycemic agents (such as metformin, sodium-glucose cotransporter 2 (SGLT2) inhibitors, glucagon-like peptide-1 (GLP-1) receptor agonists), or insulin therapy. Metformin, GLP-1 receptor agonists and other drugs can not only reduce blood glucose, but also improve systemic and vascular insulin sensitivity, which is helpful to partially restore the damaged VEGF signaling pathway and create conditions for vascular repair. One of the potential mechanisms of accelerated AS and oxidative stress in skeletal muscle in PAD is the increase of ROS (52). NADPH oxidase (NOX) and mitochondria are major contributors to ROS production in blood vessels (53). Thus, maintaining intact function of intravascular mitochondria is required for regulating ROS production and nonbioavailability, and mitochondria may be a novel therapeutic target for improving lower limb vascular function, O_2_ transfer, and functional capacity, ultimately reducing leg pain and improving quality of life in PAD patients. Current and emerging approaches focus on increasing ATP synthesis, reducing or scavenging ROS, reducing permeability transition events, and inducing mitochondrial biogenesis. Several naturally occurring substances, including co-enzyme Q, idebenone, and dimethylglycine, have been demonstrated to improve mitochondrial ATP synthesis in patients with various mitochondrial disorders (54). Additional compounds have been generated to reduce ROS production or directly scavenge ROS to reduce the burden of oxidative stress associated with mitochondrial pathologies (55). Given the apparent presence of oxidative stress and oxidative phosphorylation damage in human PAD skeletal muscle, mitochondrial targeted therapy may remain a therapeutic area that has not been fully explored. Recent preclinical evidence suggests that antioxidant therapy shows promise in both chronic ischemia and acute ischemia-reperfusion models. Acute mitochondrial antioxidant (MitoQ) intake improves endothelial function, antioxidant enzyme activity, and exercise tolerance in patients with PAD (56, 57). Lejay et al. reported that N-acetylcysteine, a global antioxidant and precursor to glutathione, improved mitochondrial respiratory function and reduced oxidative stress in mice subjected to chronic severe ischemia in both normal Swiss mice and apolipoprotein E-deficient mice (58, 59). Similarly, treatment using cardiolipin targeting the tetrapeptide elanfertide has also been shown to ameliorate ischemic myopathy in mice with critical limb ischemia (CLI) (60). Another study employed a transgenic mouse overexpressing a mitochondrial-targeted catalase (scavenger of hydrogen peroxide), which was shown to decrease ischemia muscle injury and improve mitochondrial function in diet-induced diabetic mice (61).

Mitochondrial targeted therapy and metabolic regulation lay the energy and REDOX foundation for cell repair. However, to achieve a fundamental remodeling of the diabetic PAD microenvironment, treatment must be multi-proned and synergistic. Directly inhibiting the production of glycotoxic end products or blocking their pathological signaling is another key pillar in parallel with the above-mentioned strategies. This aims to mitigate the persistent direct vascular damage of hyperglycemia at its source. In the context of PAD, the intervention against the toxicity of AGEs can be divided into two main lines: “reducing the generation” and “blocking the signal”. The first is the inhibitor of AGEs formation, which reduces the formation of AGEs.Pyridoxamine is a vitamin B6 derivative that binds to reactive carbonyl groups and Amadori intermediates, and has both metal chelation and antioxidant effects. Pyridoxamine treatment ameliorates large artery stiffening and cerebral artery endothelial dysfunction in old mice (62). Benfothiamine is a lipid-soluble thiamine prodrug, which activates transketolase, transfers glycolytic intermediates into the pentose phosphate pathway, and reduces the formation of AGEs precursors. It can reduce skin autofluorescent AGEs and improve endothelial function in diabetic neuropathy (63). Animal experiments show that anti-RAGE mAb can reverse the reduction of blood flow in the hind limbs of diabetic mice and increase endothelium-dependent vasodilation. There is direct evidence of improved blood flow and reduced inflammatory infiltration in the PAD model (64). Rage-diaph1 interaction inhibitor blocked the binding of RAGE to Diaph1 and inhibited Rac1-NF-κB signaling. Foam cell formation of macrophages was reduced and IL-10 was restored in mice, suggesting a beneficial effect on AS and inflammatory environment of PAD (65). RAGE229, a small molecule RAGE antagonist, can increase the wound closure rate by about 30% on day 21 in diabetic mice with hind foot ischemia-reperfusion injury, and simultaneously show a reduction in infarct size and neutrophil infiltration in myocardial I/R and DTH inflammation models, suggesting that RAGE229 is also effective in limb ischemia-reperfusion injury (66). In addition, FPS-ZM1 and TTP488 (Azeliragon) also have potential in the treatment of PAD (67).

## 4 Pro-angiogenic and tissue regenerative strategies

Bioendogenous processes including angiogenesis, arteriogenesis, and angiogenesis contribute to tissue repair but are often insufficient in CLI. This limitation indicates the need to develop therapeutic interventions to enhance these natural repair mechanisms. Currently, a variety of genes, cells, proteins, peptides, and metal ions are being explored to activate vascularization activity in outer nuclear cells. Each approach targets angiogenesis through a different mechanism, such as up-regulation of proangiogenic factors, use of cells to promote angiogenesis, or modification of the ischemic microenvironment. Despite their potential, these strategies also face challenges, including selective targeting of ischemic tissue, maintenance of long-term efficacy, and controlled release of bioactive molecules. Biomaterials have emerged as versatile platforms to address these barriers, improving the stability, bioactivity, and targeting precision of therapeutics (68, 69). These approaches can be broadly categorized into three synergistic fronts (1): the selective delivery and spatiotemporal control of growth factors (e.g., VEGF) to effectively stimulate angiogenesis (2); the application of cell-based therapies (e.g., stem cells) or their derived exosomes, which exert combined pro-angiogenic and immunomodulatory effects; and (3) the use of advanced biomaterial systems (e.g., scaffolds, hydrogels) as engineered platforms to localize and enhance the efficacy of therapeutic agents.

### 4.1 Precise regulation strategy based on growth factors

The following growth factors have been shown to have pro-angiogenic potential, especially important in PAD patients: VEGF, isomerism: VEGF-A-E); Placental growth factor (PLGF, isoforms: PLGF-1 and-2); Fibroblast growth factors (FGF-1, isomerism: FGF-1 to FGF-23, class 23); Hepatocyte growth factor (HGF, isomorphs HGF/natural killer (NK)1 and HGF/NK2); Platelet-derived growth factor (PDGF, isomers: PDGF-A to D; five homologues/heterodimers: PDGF-AA, -AB, -BB, -CC and-DD); “Angiopoietin (Ang, isomers: Ang-1, -2, -3, and -4) and erythropoietin (EPO) (70). Growth factors can be delivered directly to target tissues as recombinant proteins (carriers such as nanoparticles are also used). The increase of local growth factors can also be achieved indirectly by substances that promote the expression of their genes, such as transcription factors, viral vectors, plasmids encoding DNA, or cellular synthesis of these factors (71).

Simple protein injection is prone to rapid degradation and lack of targeting. Current research focuses on using hydrogels, microspheres, or nanoparticles as controlled-release carriers to deliver vascular endothelial growth factor, basic fibroblast growth factor, and other factors to the ischemic site for long-term and localized delivery, and simulate their physiological release curves to generate more mature and stable functional blood vessels. It has been reported that polylactic acid co-glycolic acid microspheres were used to control the release of vascular endothelial growth factor. VEGF-microspheres were embedded into polycaprolactone fumarate (PCLF) nerve conduits to bridge 1 cm sciatic nerve defects in rats. It was confirmed that the microspheres could maintain VEGF bioactivity and induce functional blood vessels *in vivo* for a long time (72). Another study found that VEGF and IGF delivered by alginate saline gel promoted stable perfusion recovery of ischemic hind limbs in aged mice and rabbits, achieving dual-factor sequential release. VEGF and IGF-1 were co-embedded, and the release lasted for 14 days without burst release. In aged mice (20 months), the blood flow of ischemic hind limbs could be restored significantly after a single injection, and the capillary density was 1.8 times higher than that of the blank gel group. In a New Zealand rabbit model of chronic ischemia, administration of the gel at a dose 2 orders of magnitude lower than that of the conventional “bare protein” regimen improved angiographic scores and perfusion pressure (73). In addition, the dynamic network of black phosphorus nanosheets and DNA hydrogel was constructed to achieve mechanical support and long-term sustained release by covalent grafting of VEGF. Superparamagnetic Fe_3_O_4_ -loaded BMP-2 was incorporated into the gel and guided by an external magnetic field to form a spatial concentration gradient, which programmed the directional growth of blood vessels and bone tissue. Furthermore, near-infrared light-responsive hollow gold nanoparticles loaded with BMP-2 were designed to trigger an “on-demand” burst release of growth factors by an external 808 nm laser irradiation (e.g., 5 minutes), and the release returned to the baseline level after the light source was turned off, thereby achieving a “space-time switch” precise regulation of angiogenesis and repair (74–77).

### 4.2 Cellular and extracellular vesicle therapy

Patients with severe PAD who are not candidates for revascularization face a poor prognosis; however, cell therapy using peripheral blood-derived or bone marrow-derived mononuclear cells, mesenchymal stem cells, or angiogenic marker-specific subsets of bone marrow cells may offer hope for these no-option PAD patients, as injected cells can enhance local angiogenesis—either by promoting the maturation of endothelial progenitors or through the secretion of angiogenic mediators—while also transducing cytoprotective signals that help preserve tissue structure (78). Cell therapy not only provides “seed cells” for neovascularization, but also dynamically regulates and repairs the microenvironment through powerful paracrine functions. A meta-analysis suggests that in patients with PAD or CLI who are not candidates for surgical or percutaneous revascularization, autologous cell therapy may reduce the risk of major amputation by 36% and increase the likelihood of wound healing by 59% (79). Additionally, autologous implantation of stem cells improved ulcer healing rates, ABI, transcutaneous oxygen tension (TcO_2_), peak walking distance (PFWD), and reduced rest pain scores compared to standard care or conventional treatment (80).

Cell-based therapies can be classified into two types based on the source of the cells used: allogeneic therapies, which utilize cells derived from healthy donors, and autologous therapies, which employ cells harvested directly from the patient (81). Some of the stem cells types used in the technique include bone-marrow mononuclear cells (BM-MNC), Granulocyte colony stimulating factor (G-CSF) mobilized peripheral blood mononuclear cells(PB-MNC), CD-34-positive or VEGF receptor-2-positive bone marrow derived EPCs and mesenchymal stem cells (MSCs) (82). A clinical trial conducted subcutaneously injected recombinant human GM-CSF to mobilize PB-MNCs and A phase I trial conducted by Murphy et al. found that intramuscularly-injected mobilized PB-MNCs or autologous BM-MNCs improved ABI and blood perfusion in CLI patients (81). Phase I/II trials revealed that intramuscular injection of EPCs including CD34^+^, CD133^+^or ALDH^br^ cells for CLI may be safe, feasible and effective. EPCs can develop into endothelial cells and then promote revascularization (79). The mechanism of EPC-mediated neovascularization involves the secretion of paracrine factors such as VEGF-A, VEGF-B, SDF-1, and IGF-1 by EPCs; these paracrine effects inhibit cell death, enhance cell proliferation, activate resident stem/progenitor cells in ischemic tissue, and recruit additional stem/progenitor cells to the ischemic site (83). The mechanism of action of MSCs in promoting neovascularization is primarily attributed to their release of cytokines, which exert paracrine or distant tissue effects that stimulate endogenous ECs to migrate, differentiate, and proliferate *in situ*, thereby replenishing depleted resident ECs. Numerous animal studies have demonstrated the benefits of MSC transplantation in ischemic conditions, and current research is exploring combination cell therapies that utilize approaches such as endothelial EPCs plus MSCs or MNCs plus MSCs (71).

One novel approach to cell therapy for PAD is to generate therapeutic cells using autologous skin fibroblasts. In response to retroviral vectors encoding the master regulators of pluripotency, SRY (sex-determining region Y)-box2 (Sox2), KLF4, and cellular myelocytomatosis (cMyc), the fibroblasts could become induced pluripotent stem cells (iPSCs). iPSC-derived ECs manifest all of the expected characteristics and functions, expressing endothelial surface markers (e.g. VE-cadherin), generating NO and angiogenic cytokines, and forming vascular networks in Matrigel. Moreover, the cell therapy could increase the perfusion of the ischemic hind limb in a mouse model of HLI (84).

Exosomes play a critical role in vascularization therapy for PAD. These 30–150 nm extracellular vesicles, released through the fusion of multivesicular bodies (MVBs) with plasma membrane, transport various biomolecules such as proteins, mRNAs, and microRNAs, which influence the behavior of adjacent and distant cells. Exosomes have demonstrated both pro-and anti-angiogenic potential depending on the microenvironment (85). In addition, some preclinical studies have shown the efficacy of exosomes in improving vascular function in PAD (**Table 2**). It is important to note that while exosome therapies show immense potential, direct *in vivo* evidence using specific PAD models (such as hindlimb ischemia) remains relatively limited (**Table 2**, Part A). Many current findings are derived from systemic atherosclerosis proxies (e.g., ApoE^-^/^-^ mice) or *in vitro* endothelial models (e.g., HUVECs and coronary-derived HCAECs) (**Table 2**, Part B). Therefore, therapeutic mechanisms extrapolated from these coronary and systemic atherosclerosis studies should be regarded as highly plausible mechanistic hypotheses for PAD, rather than fully established clinical facts. Future investigations must prioritize definitive PAD ischemia models to strictly validate these pathways.

**Table 2:** Different types of exosomes for improving PAD vascular function.

Part A: Direct PAD & Ischemia/wound healing models (*In Vivo* evidence)				
Source of exosomes	Type	miRNAs involved	Model	Effect
MSC	hucb-MSCs	miR-21-5p	Vascular endothelial tip cells	Promote vascular endothelial tip cell proliferation and migration by downregulating TGF-β1 (86)
EPC	hUCB-EPCs	miR-145	Mouse hind lower limb ischemia model	Promote therapeutic neovascularization via miR-145/SIRT1 pathway (87)
PC	mouse brain microvascular pericyte	miR-210-5p	Model of secondary vascular ischemia	Target ISCU1/2 can stabilize HIF-1α, improve mitochondrial membrane potential and inhibit lipid peroxidation, and indirectly improve local vascular survival (88)

Part B: Atherosclerosis proxies & mechanistic analogies (*In Vitro* & Systemic AS Models)				
Source of exosomes	Type	miRNAs involved	Model	Effect
MSC	BM-MSCs	miR-210	HUVECs	Promote angiogenesis (89)
MSC	BM-MSCs	miR-9	HUVECs	Promote angiogenesis (89)
MSC	BM-MSCs	miR-105	HUVECs	Promote angiogenesis (89)
MSC	BM-MSCs	miR-126	HUVECs	Promote angiogenesis (89)
MSC	BM-MSCs	miR-135b	HUVECs	Promote angiogenesis (89)
MSC	ASC	miR-146a	HUVECs;	Promote angiogenesis (90)
MSC	MenSCs	miR-574-5p	ApoE^-^/^-^atherosclerotic mouse model	Alleviate AS through the NF-κB signaling pathway (91)
MSC	BM-MSCs	miR-21a-5p	ApoE^-^/^-^ atherosclerotic mouse model	Promote M2 macrophage polarization and reduces macrophage infiltration to attenuate atherosclerosis (92)
MSC	huc-MSCs	miR-143-3p	HCAECs	Delay endothelial cell senescence through targeting COX-2 (93)
MSC	huc-MSCs	miR-100-5p	ApoE^-^/^-^ atherosclerotic mouse model	It can inhibit eosinophil development and inflammatory response through the FZD5/Wnt/β-catenin pathway, thereby alleviating atherosclerosis (94)
MSC	BM-MSCs	miR-let7	ApoE^-^/^-^atherosclerotic mouse model	Promote M2 macrophage polarization in the plaque through miR-let7/HMGA2/NF-κB pathway (95)
EPC	PB-ECFCs	miR-21-5p	Rat model of AS	Regulate autophagic flux to promote vascular endothelial repair by inhibiting SIPL1A2 in atherosclerosis (96)
EPC	mouse umbilical cord blood–derived CD133^+^ endothelial progenitor cells	miR-199a-3p	ApoE^-^/^-^ atherosclerotic mouse model	Inhibite iron denudation in endothelial cells and alleviated AS through miR-199a-3p/SP1 axis (97)

MSC, Mesenchymal Stromal Cells; hucb-MSCs, Human Umbilical Cord Blood Mesenchymal Stem Cells; TGF-β1, Transforming Growth Factor β1; HUVEC, Human Umbilical Vein Endothelial Cells; BM-MSCs, Bone Marrow-Derived MSCs; ASC, Adipose-Derived Mesenchymal Stem Cells; MenSCs, Menstrual Blood-Derived Mesenchymal Stem Cells; ApoE−/−, Apolipoprotein E Knockout; AS, Atherosclerosis; NF-κB, Nuclear Factor-Kappa B; M2, Alternatively Activated; huc-MSCs, Human Umbilical Cord Mesenchymal Stem Cells; HCAECs, Human Coronary Artery Endothelial Cells; COX-2, Cyclooxygenase-2; FZD5, Frizzled Class Receptor 5; HMGA2, High Mobility Group AT-Hook 2; EPC, Endothelial Progenitor Cells; PB-ECFCs, Human Peripheral Blood–Derived Endothelial Colony-Forming Cells; SIPA1L2, Signal-Induced Proliferation-Associated 1 Like 2; hUCB-EPCs, Human Umbilical Cord Blood–Derived Endothelial Progenitor Cells; SIRT1, Silent Information Regulator 1; SP1, Sp1 Transcription Factor; PC, Pericytes; ISCU1/2, ISCU Isoform 1/Cytosolic ISCU/ISCU Isoform 2/Mitochondrial ISCU; HIF-1α, Hypoxia Inducible Factor 1α; miRNA, MicroRNA.

### 4.3 Biomaterials and engineered delivery systems

With advancements in cell biology, materials science, and regenerative medicine, utilizing nanobiomedical technologies to construct scaffolds capable of carrying cells, growth factors, and drugs—such as hydrogels, nanoparticles (NPs), and nanospheres—has emerged as a new approach for treating PAD. Biomaterials possess excellent biocompatibility and support cell adhesion, delivery, and growth. Furthermore, as scaffolds, biomaterials can locally deliver drug payloads to the injury site, enhancing drug utilization.

Hydrogels are generally considered hydrophilic gels with a 3D network structure, which can swell promptly after absorbing water and keeping a large amount of water in a swollen state without dissolving. The water absorption of hydrogels is usually negatively correlated with the degree of crosslinking, which means the higher the degree of crosslinking, the lower the water absorption. The materials used to prepare hydrogels usually have excellent biodegradability and designable mechanical properties. Researchers tend to prepare hydrogels with mechanical properties similar to the natural soft tissues to attenuate the inflammation of neighboring cells (98). Since then, the biomedical potential of hydrogels has been increasingly exploited for drug delivery, tissue engineering, and 3D cell culture (99). Conventional strategies for CLI are often hampered by limited revascularisation efficacy and the transient bioavailability of pro-angiogenic agents. Hydrogel-based therapeutic angiogenesis—an emerging interdisciplinary paradigm—has therefore attracted intense interest as a means to overcome these drawbacks. Depending on the origin of the constituent polymers, hydrogels can be broadly classified as natural, synthetic, or hybrid systems, each of which can be used individually or in combination to fabricate matrices tailored to CLI therapy. Over the past decade, a diverse array of hydrogel platforms has been developed to promote sustained neovessel formation, orchestrate immune modulation, and enhance tissue perfusion in ischaemic limbs (100).

In addition to hydrogels, there are still many other types of biomaterials that have been studied to treat PAD, such as scaffold, nanoparticle (NP) and spheroid (101–103). Recently, Mu et al. fabricated an electroglucomannan decanoate (GMDE) scaffold to mimic microbial invasion and isolate endogenous galectin-1. *In vivo* results showed that GMDE scaffolds could effectively promote blood perfusion (104). Li et al. designed a nanoparticulate anchored hydrogel scaffold and evaluated its therapeutic effect on ischemic hind limbs in mice. *In vivo* results showed that the stent could significantly promote the restoration of blood perfusion (105).

NPs have emerged as a new model of drug delivery system with advantages such as targeting and slow release. According to several studies, cerium oxide nanoparticles (CNPS) have shown therapeutic effects *in vivo* as well as *in vitro (*106, 107). Park et al. confirmed the angiogenic properties of CNP in a mouse model of hindlimb ischemia (108). Patients with PAD have typical ischemic myopathy in the legs. Myopathy is histologically characterized by progressive muscle fiber degeneration and fibrosis with increased oxidative damage (109). PAD induces tissue ischemia and ischemia/reperfusion states, resulting in ROS production at levels well above the cellular antioxidant potential. Poly (propylene sulfide) (PPS) is a hydrophobic polymer that is sensitive to ROS. Upon exposure to ROS, the morphology changes to the more hydrophilic poly (propylene sulfonate oxide) and poly (propylene sulfonate). More recently, O’Grady et al. have explored PPS as a stand-alone, locally sustained antioxidant therapy. The investigators found that PPS particles functionally improved recovery from hindlimb ischemia by increasing foot pad hemoglobin saturation and perfusion by approximately 15 to 25%, as well as early remodeling of proximal limb vessels (110).

The therapeutic benefit of single cell transplantation is not ideal. However, when the cells become spheres, the efficiency could be significantly improved, because the 3D sphere system can create a more biomimetic microenvironment (111). Therefore, cell spheres capable of synergize with biomaterials have been extensively studied. Choi et al. cultured human adipose-derived stem cells (hASCs) in FGF-2 matrices, which in turn developed into augmented cell spheres (FECs-Ad) and were capable of secreting multiple angiogenic growth factors. Their study proved that FECs-Ad injection promoted angiogenesis in the ischemic hind limb while increasing VEGF expression (103).

## 5 Toward a new paradigm: multimodal integration for systemic vascular repair

### 5.1 Dual-modal immune regulation: constructing repair homeostasis after revascularization

The comprehensive strategy of modern revascularization goes far beyond mechanical recanalization. One of the central challenges is how to manage the systemic inflammatory response induced by revascularization itself, with activation of the NLRP3 inflammasome as a key pathway. This response is an important driver of intimal hyperplasia, neoatherosclerosis and even in-stent restenosis. Therefore, the postoperative treatment paradigm must evolve from the traditional ‘antithrombotic’ leading to a new model of ‘antithrombotic and anti-inflammatory’. This constitutes the first pillar of our comprehensive multimodal approach: revascularization with postoperative systemic antiinflammatory modulation.

#### 5.1.1 Systemic anti-inflammation therapy with low-dose colchicine

Low-dose colchicine inhibits microtubule assembly and blocks NLRP3 inflammasome activation. Large-scale clinical trials, such as COLCOT and LoDoCo2, have shown that low-dose colchicine reduces major cardiovascular events in patients with coronary artery disease (CAD). However, these trials were conducted exclusively in CAD populations, not in PAD cohorts. If validated in future PAD-specific trials, colchicine could become part of postoperative management. Until then, its efficacy in improving peripheral vascular patency remains an extrapolation from coronary evidence (112, 113). Colchicine was originally an anti-inflammatory drug extracted from autumn saffron and used by the ancient Greeks and Egyptians. The anti-inflammatory mechanism of colchicine mainly includes the following aspects (1). Colchicine has a high affinity for specific domains of β-tubulin, resulting in (i) inhibition of tubulin assembly into MT, (ii) disassembly of formed tubulin, and/or (iii) inhibition of membrane-bound tubulin-sensitive cellular processes. Colchicine can inhibit NLRP3 inflammasomes present in myeloid cells, including monocytes, neutrophils, and eosinophils, or its downstream inflammatory cytokine activation. The highly mature inflammatory cytokines IL-1β and IL-18 produced by NLRP3 can lead to the progression of atherosclerotic plaques and even plaque shedding (114). Colchicine has been tested in coronary populations. A randomized trial in diabetic patients undergoing bare-metal stent implantation showed that colchicine reduced angiographic restenosis (16% vs 33%; OR 0.38, 95% CI 0.18–0.79, p = 0.007) (115). The LoDoCo trial showed that low-dose colchicine reduced major adverse cardiovascular events in patients with chronic coronary disease (116, 117). No randomized trial has evaluated colchicine after PAD revascularization; its use in PAD is therefore a hypothesis-generating extrapolation from coronary evidence.

#### 5.1.2 Remodeling of local immune microenvironment:

Direct targeting by immunomodulatory biomaterials. However, systemic drugs are difficult to precisely regulate the complex immune microenvironment in the ischemic region. Genetic engineering methods have been used to solve this problem (118). For example, Jiang et al. genetically engineered ADscs to over-express the estin-181 protein, a protein normally associated with neurodevelopment that has been shown to improve endothelial cell survival 102 and promote an anti-inflammatory macrophage M2 phenotype. The goal of their study was to reduce restenosis after angioplasty, and the retention of M2 macrophages is essential to achieve this goal, as they help remove senescent cells known to contribute to restenosis. After implantation of the hydrogel into mice, expression of the proinflammatory markers TNF-α and IL-6 associated with M1 macrophages was reduced by more than 50%, while expression of the autoinflammatory markers Arg-1 and IL-10 associated with M2 macrophages was increased four-fold (119, 120). Even in the absence of transplanted cells, biomaterials can still exert immunomodulation on ischemic muscle tissue by releasing anti-inflammatory factors. This strategy has the potential to avoid genetic modification of cells. A potential advantage of using biomaterials to deliver anti-inflammatory factors is the greater control of release kinetics compared to conventional delivery of saline-soluble factor enrichment. For example,.Kwee et al. developed a method in which they implanted antigen-releasing scaffolds in animals previously vaccinated with the same antigen. Hindlimb ischemic mice previously vaccinated with ovalbumin (OVA) and aluminum vaccines subsequently received OVA-releasing scaffolds. This implantation results in the accumulation of antigen-specific TH2 T cells at the ischemic site of the limb, thereby promoting restoration of blood perfusion in the ischemic tissue. These examples illustrate how biomaterial based delivery of immunomodulatory factors can affect functional recovery in ischemic limb muscles (121). Immunomodulatory biomaterials continuously release immunomodulatory signals locally. Or directly talk to immune cells through its material properties (such as topology, mechanical properties, surface chemistry), so as to actively remodel ischemic tissues from pro-inflammatory microenvironment into a pro-regenerative niche suitable for angiogenesis (118).

This combination represents a change from systemic anti-inflammatory treatment alone toward combined systemic and local immune modulation. Low-dose colchicine may suppress NLRP3-mediated chronic cardiovascular inflammation, while locally implanted immunomodulatory biomaterials may modify the ischemic microenvironment through their physicochemical properties and drug-release profile. The two approaches differ in route and target, and whether their combination improves long-term vascular patency or tissue regeneration in PAD has not been tested.

### 5.2 Spatiotemporally controlled delivery: precision regenerative therapy led by biomaterials

Therapeutic angiogenesis with direct delivery of growth factors holds great promise for treating patients with leg ischemia, but current methods have not found success in clinical trials. There are several major hurdles, including short half-lives of growth factors. In addition, long-term disease states and co-morbidities, such as diabetes and obesity, make patients resistant to angiogenic stimuli. various types of biomaterials have been used to deliver growth factors for PAD treatment. Incorporation of these signaling molecules into biomaterials, as opposed to a bolus injection, protects factors from rapid enzymatic degradation, resulting in sustained release over a longer therapeutic window (122, 123).

With the boosting studies of biomaterials, more and more research is focusing on the combination of biomaterials with growth factors and stem cells. Among all these materials, hydrogel is frequently used due to its 3D porous structure and efficient water content. Thus, hydrogels are chosen as carriers for growth factors and stem cells to treat PAD (**Figure 6**). Stem cell therapy has been shown to treat limb ischemia but is still limited by problems such as inaccurate cell release and low cell survival (124). Encapsulation of cells in injectable hydrogels or other tissue engineered scaffolds that provide mechanical protection against cell membrane damage as the syringe flows has been shown to address these challenges. For example, researchers have created a shear-thinning, injectable recombinant hydrogel to enhance the survival of encapsulated induced pluripotent stem cell-derived endothelial cells in murine PAD models (125). The material is built from multi-arm poly (ethylene glycol) functionalized with cell-adhesion peptides and thermo-responsive poly(N-isopropylacrylamide) (PNIPAM). Under high shear it thins to 0.5–2 Pa·s, yet at low shear it self-heals to 20–100 Pa·s within one second. PNIPAM’s lower critical solution temperature of 32 °C provides a secondary, *in situ* crosslinking that stiffens the network, boosting cell retention, proliferation, and secretion of pro-vascular factors. To further reduce the mechanical stress imposed during injection, a dynamically responsive hydrogel inspired by laminin was engineered. Functionalization with the laminin-derived peptide Fmoc-DDIKVAV enables the hydrogel to self-assemble into a nanofibrous scaffold that instantaneously provides a protective sanctuary, shielding encapsulated cells from the mechanical damage associated with droplet formation and shear forces at the needle outlet (126). Mechanistic analyses revealed that cell survival is governed primarily by the fluid-mechanical events of droplet generation rather than by the magnitude of the shear rate itself, with the threshold droplet behavior serving as a reliable predictor of the observed viability. In a further advance, non-Newtonian polymer–nanoparticle hydrogels were exploited to safeguard cell viability during injection (127). Prepared by combining dodecyl-modified hydroxypropyl methylcellulose (HPMC-C_12_) with PEG-b-PLA nanoparticles, the formulations markedly reduced the injection pressure and limited cellular damage by maintaining low shear stress throughout the injection process. Consequently, viabilities of multiple cell types—including MSCs—remained consistently ≥86% when delivered within the hydrogel, compared with 68% observed for PBS injection alone (69). Due to the lack of blood perfusion, the temperature of the ischemic limb is usually lower than that of normal tissue (128). Inspired by this, Wang et al. developed an Mc-based temperature-responsive hydrogel coated with human placental mesenchymal stem cells (PMSCS). The results showed that the Mc-based hydrogel could effectively release PMSCs into the ischemic area, thereby improving blood perfusion and promoting revascularization (129).

**Figure 6:**
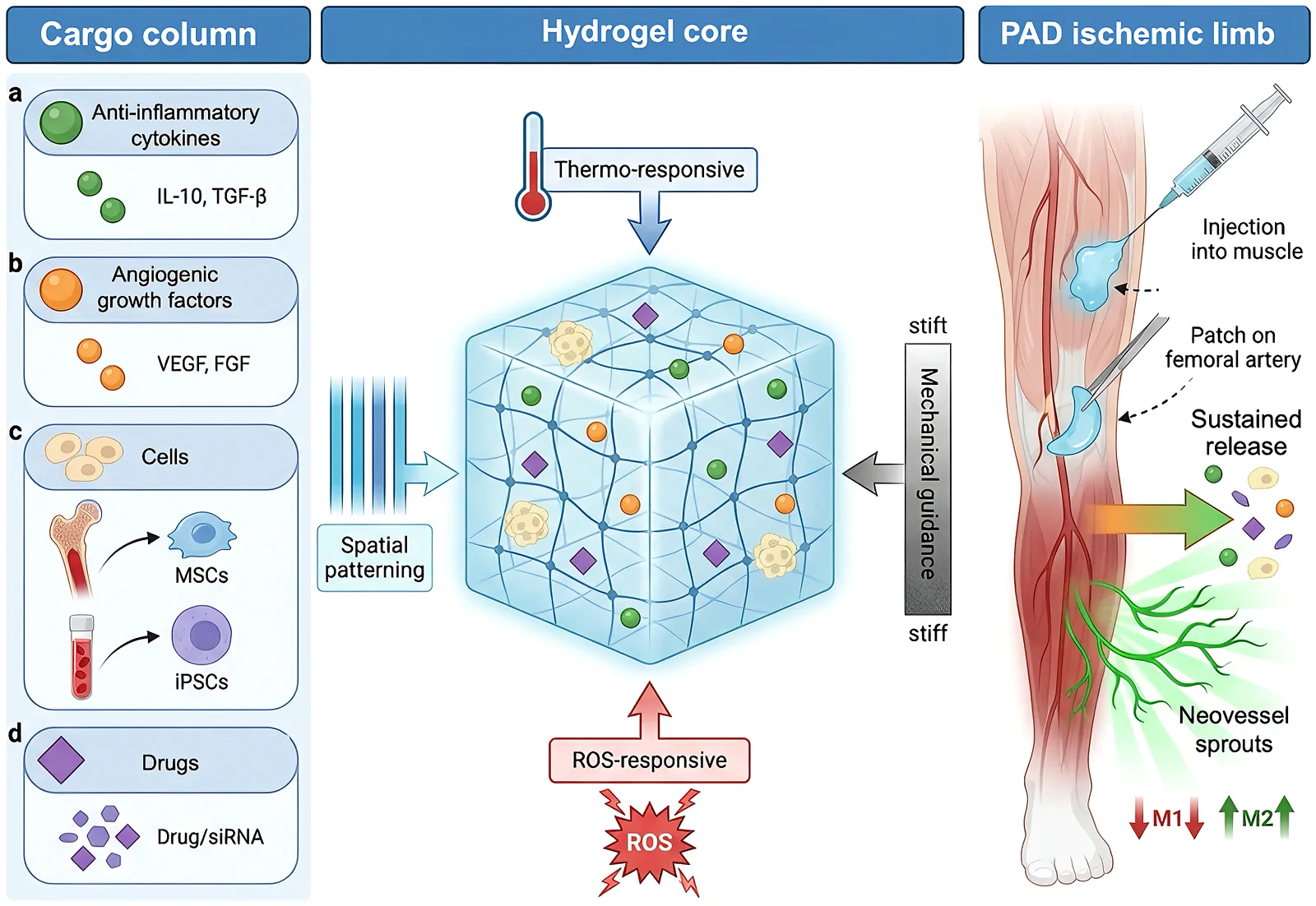
Schematic illustration of the multi-responsive hydrogel system for PAD treatment. The system comprises a cargo column, a hydrogel core, and application in the PAD ischemic limb. **(a)** Anti-inflammatory cytokines (IL-10, TGF-β). **(b)** Angiogenic growth factors (VEGF, FGF). **(c)** Cells, including mesenchymal stem cells (MSCs) and induced pluripotent stem cells (iPSCs). **(d)** Drugs (Drug/siRNA). The hydrogel core features thermo-responsive and ROS-responsive properties, spatial patterning, and mechanical guidance. Upon injection into the muscle or application as a patch on the femoral artery, the hydrogel achieves sustained release, promotes neovessel sprouts, and facilitates macrophage polarization from M1 to M2. FGF, Fibroblast growth factor; IL-10, Interleukin-10; iPSCs, Induced pluripotent stem cells; M1, Classically activated macrophages; M2, Alternatively activated macrophages; MSCs, Mesenchymal stem cells; PAD, Peripheral artery disease; ROS, Reactive oxygen species; siRNA, Small interfering RNA; TGF-β, Transforming growth factor-beta; VEGF, Vascular endothelial growth factor.

Recent advancements in 3D bioprinting have enabled the precise fabrication of both micro-vascular networks (130) and scalable, large-diameter tissue-engineered blood vessels (131). By closely mimicking native vascular architectures to rapidly restore perfusion in ischemic tissues, these bioengineered grafts represent a versatile structural platform for future multimodal PAD treatments.

Integrating responsive cells with precisely engineered biomaterials establishes a highly controllable therapeutic system that replaces unpredictable tissue regeneration with on-demand, precision interventions—a strategy that holds promise for treating complex tissue defects such as PAD.

### 5.3 Combining the modalities: evidence of synergy and remaining gaps

While individual therapeutic approaches—such as immunomodulation, metabolic correction, or angiogenic therapy—have shown efficacy in preclinical models, the central premise of multimodal integrated therapy requires these modalities to act synergistically rather than merely in parallel. Currently, comprehensive *in vivo* data evaluating the simultaneous integration of all these modalities in PAD remain scarce. Emerging evidence from bioengineering and PAD-relevant preclinical models supports the potential of combined approaches, although formal synergy testing remains rare. For example, in a PAD-relevant hindlimb ischemia model, sequential release of VEGF and IGF-1 from an alginate hydrogel restored perfusion in aged mice and rabbits at doses two orders of magnitude lower than single-protein regimens (73). Similarly, VEGF/SDF-1 co-delivery enhanced endothelial progenitor recruitment and ischemic recovery beyond either factor alone (122). In adjacent fields, dual-delivery scaffold systems designed to sequentially release immunomodulatory signals alongside pro-angiogenic factors have shown enhanced vascular network maturation in chronic wound models, though these findings have not been validated in PAD (132). Similarly, combining stem cell therapy with metabolic preconditioning has been proposed to improve cell survival in the ischemic niche, but direct PAD evidence remains lacking.

Despite these dual-therapy approaches, the full integration of immune, metabolic, and regenerative therapies remains largely unachieved. To our knowledge, no study has tested a regimen that combines (i) immunomodulation, (ii) metabolic correction, and (iii) tissue-engineered regeneration within a single PAD model, let alone in patients. The field must transition from parallel investigations to rigorously designed combination trials to determine whether systemic integrated repair outperforms isolated interventions.

## 6 Challenges and future perspectives

The evolving paradigm of PAD management—shifting from mechanical revascularization toward multimodal integrated therapy that combines immunomodulation, metabolic correction, and tissue regeneration—holds immense promise. However, translating these cutting-edge strategies from bench to bedside is fraught with significant challenges that must be systematically addressed to realize their full clinical potential.

### 6.1 Key challenges in translational implementation

#### 6.1.1 Biocompatibility and long-term safety of biomaterials

The clinical application of biomaterials, including hydrogels, scaffolds, and nanoparticles, faces substantial hurdles regarding long-term biocompatibility. The degradation kinetics of these materials must be precisely matched to the tissue repair timeline, as premature degradation leads to loss of therapeutic efficacy, while delayed degradation may provoke chronic foreign-body responses and sustained inflammation (69, 98). Furthermore, the immunogenicity of degradation products remains inadequately characterized, with potential risks of secondary inflammatory reactions that could paradoxically exacerbate the pro-inflammatory microenvironment that these therapies aim to resolve (118). The long-term fate of implanted materials, including their potential for systemic distribution and accumulation, requires rigorous investigation in large-animal models before clinical translation.

#### 6.1.2 Efficacy gaps in complex patient populations

Despite encouraging preclinical results, the efficacy of current immunomodulatory and regenerative strategies remains suboptimal in patients with complex comorbidities, particularly those with long-standing diabetes, chronic kidney disease, or advanced age. These conditions are associated with an intrinsically hostile microenvironment characterized by persistent hyperglycemia, elevated oxidative stress, and entrenched inflammatory states that render tissues resistant to pro-angiogenic and immunomodulatory stimuli (41, 47). The therapeutic resistance observed in these populations underscores a critical gap between proof-of-concept studies in healthy or young animal models and real-world clinical scenarios, necessitating the development of disease-specific preclinical platforms that better recapitulate human PAD pathophysiology.

#### 6.1.3 Regulatory and manufacturing bottlenecks

The regulatory pathway for combination products, such as cell-material complexes or growth factor-loaded scaffolds, remains ill-defined, creating substantial obstacles to clinical translation. Unlike conventional drugs or devices, these advanced therapeutic medicinal products require integrated evaluation of their biological, material, and mechanical components, complicating the approval process (118, 123). The absence of harmonized manufacturing standards, quality control protocols, and validated potency assays further impedes scalability and reproducibility. Additionally, the high costs associated with personalized biomaterials, cell manufacturing, and gene-editing approaches pose significant economic barriers that may limit widespread adoption, particularly in resource-limited healthcare settings.

#### 6.1.4 Patient heterogeneity and predictive biomarkers

PAD patients exhibit inter-individual variability in inflammatory status, metabolic derangements, and microenvironmental characteristics. This heterogeneity complicates the selection of optimal therapeutic strategies, as the same immunomodulatory intervention may yield divergent outcomes depending on baseline immune profiles (122, 133). Currently, reliable predictive biomarkers that can stratify patients and guide therapy selection are lacking. Traditional anatomical imaging modalities, such as computed tomography angiography (CTA) and magnetic resonance angiography (MRA), while invaluable for assessing macrovascular stenoses, are insufficient for evaluating microcirculatory perfusion, tissue viability, and the dynamic resolution of inflammation—parameters that are essential for monitoring the efficacy of immunomodulatory and regenerative therapies.

#### 6.1.5 Knowledge gaps in disease mechanisms

Despite significant advances, fundamental questions regarding the spatiotemporal dynamics of immune cell trafficking, the interplay between systemic and local inflammation, and the determinants of successful or failed tissue regeneration in PAD remain incompletely understood. The relative contributions of different immune cell subsets, the optimal timing for immunomodulatory intervention relative to revascularization, and the mechanisms governing the transition from chronic inflammation to resolution and repair are areas that require deeper mechanistic investigation.

### 6.2 Future directions: toward precision and integration

#### 6.2.1 From monotherapy to sequential, modular combination strategies

The future of PAD therapy lies not in discovering a single therapy but in designing rationally sequenced, modular combination regimens that address the multifaceted nature of the disease. Given the current lack of comprehensive combination-therapy data in PAD, we present the following clinical roadmap as a conceptual proposal for future investigation rather than an established finding. This proposed framework could logically involve (1): initial revascularization using drug-coated balloons or bioresorbable scaffolds to restore macrovascular patency; (2) subsequent local delivery of immunomodulatory signals via smart hydrogels to reprogram the lesion microenvironment from M1-dominant to M2-dominant macrophage polarization; (3) spatiotemporally controlled release of pro-angiogenic factors to stimulate neo-vessel formation; and (4) final administration of stem cells or exosomes to promote durable tissue regeneration and functional recovery. This “revascularization– immune microenvironment remodeling– structural regeneration” cascade aims to achieve synergistic rather than merely additive therapeutic effects.

#### 6.2.2 Precision medicine: patient stratification and personalized therapy

The integration of multi-omics technologies—including genomics, transcriptomics, proteomics, and metabolomics—holds considerable potential for patient stratification and personalized therapy. Comprehensive immunophenotyping and microenvironmental profiling could identify subpopulations of patients most likely to benefit from specific interventions, such as NLRP3 inflammasome inhibitors, macrophage-modulating agents, or growth factors. Concurrently, advances in 3D bioprinting and biodegradable materials are enabling the fabrication of patient-specific vascular grafts and tissue-engineered scaffolds customized to individual anatomical and pathological characteristics (131, 134). The development of companion diagnostics to guide therapy selection couldbe essential for maximizing efficacy and minimizing unnecessary exposure to costly or potentially harmful interventions.

#### 6.2.3 Advanced imaging and endpoint development

To accurately monitor treatment response and facilitate clinical trial design, novel molecular imaging techniques and tissue perfusion assessment tools must be developed and validated. These technologies should enable real-time, non-invasive evaluation of microcirculatory restoration, inflammatory resolution, and tissue viability. Furthermore, there is an urgent need for standardized, clinically meaningful endpoint indicators that capture “functional regeneration” rather than merely anatomical patency—a shift that would align clinical trial outcomes more closely with patient-relevant measures such as walking distance, quality of life, and amputation-free survival.

#### 6.2.4 Overcoming the immune-metabolic barrier

Given the central role of the immune-metabolic axis in PAD pathogenesis, future therapeutic strategies must simultaneously address both arms of this vicious cycle. Combinatorial approaches that pair metabolic correction—such as stringent glycemic control, SGLT2 inhibitors, or GLP-1 receptor agonists—with targeted immunomodulation may prove more effective than either strategy alone. The development of “dual-action” biomaterials capable of delivering both metabolic modulators and immunomodulatory signals in a coordinated manner represents an emerging research direction (132). Additionally, mitochondrial-targeted therapies, which address the intersection of metabolic dysfunction and oxidative stress, warrant further exploration as adjuncts to immunomodulatory strategies.

#### 6.2.5 Interdisciplinary collaboration and knowledge integration

The complexity of PAD pathophysiology demands deep integration across traditionally siloed disciplines, including vascular biology, immunology, metabolism, materials science, bioengineering, and clinical pharmacology. Such collaboration is essential not only for advancing mechanistic understanding but also for accelerating the translation of preclinical discoveries into clinical applications. Academic-industry partnerships and multi-stakeholder consortia couldbe instrumental in overcoming regulatory, manufacturing, and economic barriers, particularly for advanced therapeutic products.

### 6.3 Concluding remarks

The goal of PAD treatment is shifting from restoring macrovascular patency alone toward also supporting endogenous repair through immunomodulation and bioengineering. Whether such combined approaches can improve functional outcomes in muscle, nerve, and skin remains uncertain. Despite substantial challenges, progress in immunology, metabolism, and biomaterials provides a basis for testing these strategies in PAD.
